# Proteomic profiling of cerebrospinal fluid uncovers distinctive pathophysiological changes and potential biomarkers in pediatric tubercular meningitis

**DOI:** 10.3389/fcimb.2025.1662783

**Published:** 2025-10-16

**Authors:** Jing Wei, Liang Zhu, Binglin Jian, Yuncui Yu, Bing Hu, Lingyun Guo, Huili Hu, Zhenzhen Dou, Linlin Liu, Gang Liu, Peng Guo

**Affiliations:** ^1^ Clinical Research Center, Beijing Children's Hospital, Capital Medical University, National Center for Children's Health, Beijing, China; ^2^ Department of Infectious Diseases, Key Laboratory of Major Diseases in Children, Ministry of Education, Beijing Children’s Hospital, Capital Medical University, National Center for Children’s Health, Beijing, China; ^3^ Research Unit of Critical Infection in Children, Chinese Academy of Medical Sciences, Beijing, China; ^4^ Key Laboratory of Major Diseases in Children, Ministry of Education, Beijing Children's Hospital, Capital Medical University, National Center for Children's Health, Beijing, China

**Keywords:** tuberculous meningitis, cerebrospinal fluid, proteomics, different pathogenesis, biomarker

## Abstract

**Introduction:**

Tubercular meningitis (TBM) is a serious pediatric infection with high mortality rates. This research aimed to characterize the alterations in cerebrospinal fluid (CSF) proteome in TBM and to identify biomarker panels that can distinguish it from other central nervous system infections.

**Methods:**

We retrospectively analyzed the CSF proteome from 104 patients, including 7 TBM, 28 with purulent meningitis (PM), 20 with viral meningitis (VM), 9 with cryptococcus neoformans meningitis (CNM), 30 non-CNS infected controls (Ctrl), and 10 brain disease (BD) controls.

**Results:**

The TBM proteome displayed greater similarity to that of PM patients. A total of 120 cytokines and receptors were significantly dysregulated in TBM CSF. Pathway analysis indicated marked upregulation of complement activation, fibrin clot formation, and microautophagy signaling, along with significant suppression of collagen degradation in TBM. Biomarker panels were established, including F2 and TYMP to differentiate TBM from PM (AUC=0.874, 95% CI, 0.748-0.999), ENPP2 and WARS1 to differentiate TBM from VM (AUC=0.929, 95% CI, 0.929-1), F12, APOM and CD163 to differentiate TBM from CCM (AUC=0.993, 95% CI, 0.869-1), and HLA-B and MGAT1 to differentiate TBM from Ctrls (AUC=0.934, 95% CI, 0.825-1).

**Discussion:**

This research will provide a highly valuable proteomics resource for a better understanding of TBM pathogenesis, yielding insights into important differential diagnostic biomarkers and potential therapeutic targets in pediatric TBM.

## 1 Introduction

Tuberculous meningitis (TBM) is a central nervous system infection caused by Mycobacterium tuberculosis (Mtb), considered the most devastating manifestation of tuberculosis (TB), with the highest incidence in the vulnerable early childhood age group (Solomons et al., 2022). A systematic review of TBM in children reported an overall mortality risk of nearly 20%, while severe neurological morbidity is noted in more than 50% of survivors, even when treated (Chiang et al., 2014). TBM often presents with non-specific symptoms in the early stages, which are easily misdiagnosed as other meningitides, such as the purulent meningitis (PM), viral meningitis (VM), and *cryptococcus neoformans* meningitis (CCM). This causes early diagnosis challenges and delays treatment, resulting in poor outcomes like death, neurological damage, and neurocognitive disorders. Therefore, timely diagnosis and differential diagnosis of TBM are crucial to prompt anti-tuberculosis therapy.

The diagnosis of TBM in children remains a challenge. Traditional diagnostic methods, such as acid-fast staining, have low sensitivity, while Mtb culture is time-consuming, resulting in delayed diagnosis and treatment (Lin, 2024). Alarmingly, commonly employed clinical markers lack specificity, thereby increasing the risk of misdiagnosing TBM as other types of meningitis, such as viral or bacterial forms (Xing et al., 2020). Recent advancements in diagnostics, such as XpertUltra and Metagenomic Next-Generation Sequencing (mNGS), have improved sensitivity and shortened diagnostic delays (Shen et al., 2021; Li et al., 2023). However, the lowered specificity and positive predictive value compared to GeneXpert MTB/RIF raised concerns, which require prospective testing in a large population, including children (Donovan et al., 2020). Additionally, mNGS may detect contaminants, causing false positives (Zhu et al., 2022). Other CSF diagnostic method, such as the Gamma-interferon release assays (IGRAs), requires large CSF volumes (>6 mL) (Luo et al., 2020), while the Adenosine deaminase (ADA) levels in bacterial and viral meningitis complicate ADA testing interpretation in TBM due to unclear diagnostic cut-offs (Pormohammad et al., 2017). Therefore, there is an urgent need for new TBM diagnostic tools suitable for use in children.

Advances in omics technologies offer promising avenues for identifying TBM diagnostic biomarkers. Previous research used 2D-based CSF proteomics to identify 19 differential proteins with ALOX-5 as a potential marker (Kataria et al., 2011). Another research used iTRAQ-based quantitative proteomics to confirm the association of S100A8 and APOB with TBM using ELISA (Ou et al., 2013). Furthermore, another similar iTRAQ-based quantitative proteomics research identified 111 differential proteins between TBM and healthy controls, highlighting NELL2 and APOB as potential diagnostic biomarkers (Mu et al., 2015; Yang et al., 2015). However, all these studies were conducted on adult TBM patients. Since the incidence rate of TBM is higher in children than in adults (Kelekçi et al., 2014), it is essential to study the CSF proteomes in childhood TBM. Currently, various studies have identified and validated host protein biomarkers with potential as diagnostic candidates for childhood TBM (Visser et al., 2015; Manyelo et al., 2019a; Manyelo et al., 2019b; Manyelo et al., 2022)using immunoassays, highlighting the urgent need to apply a proteomic strategy to detect more proteins suitable for diagnosis in pediatric TBM patients.

In this study, we performed a retrospective analysis using an unbiased proteomics approach to investigate proteome changes among patients with TBM, PM, VM, CCM, brain disease patients (BD), and non-CNS infected controls (Ctrls). To our knowledge, there is the first CSF proteomics research in pediatric TBM patients. We uncovered intriguing disparities between TBM and other CNS-infected meningitis through functional enrichment analysis. Finally, we generated potential biomarker panels for TBM diagnosis and differentiation from other CNS infections. This research aimed to reveal distinctive changes in the CSF proteome in pediatric TBM, discover valuable CSF protein biomarkers for TBM diagnosis, and provide potential targets for TBM therapy.

## 2 Materials and methods

### 2.1 Ethical approval

This study received approval from the Ethics Committee of Beijing Children’s Hospital, Capital Medical University (Ethical approval number: [2023]-E-156-Y). All CSF samples were obtained from the biobank of Beijing Children’s Hospital. Written informed consent for participation in this study was provided by the participants’ legal guardian.

### 2.2 TBM patients and CSF samples

A total of 104 CSF archived samples from hospitalized patients were analyzed. Patients enrolled at Beijing Children’s Hospital (National Children’s Medical Center) between December 2016 and March 2021, with a clinical suspicion of meningitis who underwent a lumbar puncture (LP) and had CSF samples collected, were included. Patients were categorized into the following groups: TBM, PM, VM, CCM, BD, and Ctrl. Patients with febrile convulsions, traumatic brain injury, and without etiological evidence were excluded. The inclusion criteria for the four types of meningitis required the isolation of a pathogen from CSF or blood culture, or the detection of the virus using PCR. Finally, 7 TBM, 28 PM, 20 VM, 9 CCM, 10 BD, and 30 Ctrls were enrolled in this research. All the patients were negative for HIV infections.

Each CSF sample was collected using a syringe and placed into a polypropylene sample collection tube. The total volume of CSF (approximately 1–2 ml) was retained, then centrifuged at 4°C for 10 minutes at 2,000 × g. Then, the supernatant was removed and stored at -80°C until analysis. Before proteomic analysis, the TBM samples were filtered by 0.22μm sterilizing filter and processed in a CL2 lab with CL3 standards to ensure biosafety. Clinical data were collected retrospectively from the patient’s medical records by clinicians.

### 2.3 Sample preparation

Protein digestion was carried out using the filter-aided sample preparation (FASP) method with minor modifications (Wiśniewski et al., 2009). Briefly, 100 μL of each CSF sample was reduced with 20 mM dithiothreitol (DTT) at 95°C for 5 minutes, followed by alkylation with 50 mM iodoacetamide (IAA) at room temperature for 45 minutes in the dark. Next, the samples were transferred to 30-KD ultrafiltration filters and centrifuged at 14,000 × g for 15 minutes at 4°C. Then, the samples were washed three times with 25 mM ammonium bicarbonate (ABC). The treated samples were digested with trypsin (enzyme-to-protein ratio of 1:50) in 25 mM ABC and incubated at 37°C for 14 hours. Finally, the digested peptides were collected after centrifugation at 14,000 g for 20 minutes. The peptide concentration was quantified using the BCA method and loaded with an equal amount for LC-MS/MS analysis.

### 2.4 LC-MS/MS analysis

LC-MS/MS analysis was conducted on an UltiMate 3000 coupled to an Exploris 480 Orbitrap mass spectrometer using an electrospray ion source (all Thermo Fisher Scientific). Purified peptides were separated at 60°C on 50 cm columns with an inner diameter of 75 µm C18 analytical column (Omitech). Mobile phases A and B consisted of 99.9/0.1% water/formic acid (v/v) and 80/20/0.1% acetonitrile/water/formic acid (v/v/v). The flow rate was maintained at 1500 nl/min, with the initial concentration of 5% B being increased linearly to 30% B over 20 minutes, followed by a further increase to 90% within 2.5 minutes, and a 2.5-minute plateau at the end.

MS data were acquired using the data-independent acquisition (DIA) scan mode for single-shot patient samples. The DIA method employed a variable isolation window of 60 windows for acquisition (**Supplementary Table S1**). A full MS scan was performed within the m/z range of 350-1,200 at a resolution of 120,000, while the DIA scan was acquired at a resolution of 30,000. The automatic gain control (AGC) target was set to a custom value. The maximum injection time was 50 ms. The high-energy collision dissociation (HCD) energy was set to 30%.

### 2.5 Mass spectrometry data processing

Raw data generated by MS underwent quantitative analysis using Spectronaut Pulsar (v18.6, Biognosys AG) with a direct DIA analysis strategy. The database searched was the SwissProt human database (released in March 2024, containing 20,433 sequences). Searches utilized carbamidomethylation as a fixed modification, acetylation of the protein N-terminus as a variable modification, and oxidation of methionines as another variable modification. The Trypsin/P proteolytic cleavage rule permitted a maximum of 2 missed cleavages. The FDR for PSM, peptides, and protein groups was set at 0.01. DIA analysis was performed with parameters set as follows: the precision iRT was calibrated based on local non-linear regression; the Q-value cutoff for precursors and proteins was 0.01; the protein label-free quantification (LFQ) method was MaxLFQ; cross-run normalization was carried out using a local normalization strategy to adjust for the systematic variance of LC-MS performance (Callister et al., 2006); the protein inference algorithm was IDPicker (Zhang et al., 2007); and the quantification MS level was MS1.

### 2.6 Statistical analysis

The pre-processing of the proteomic data was performed using the wkomics (https://www.omicsolution.com/wkomics/main/) analysis platform. Proteins with a missing ratio ≥ 40% across all three groups (Meningitis, BD, and Ctrl) were removed. The remaining proteins, which still had a missing ratio of ≥ 40% in each group, were imputed with the minimum value for that group. Proteins with a missing ratio < 40% in each group were imputed by the Sequential K-Nearest Neighbors (SeqKNN) method (Kim et al., 2004). The imputation method was validated by NAguiderR to evaluate method priority (Wang et al., 2020).

The abundance of a protein in each sample was normalized to its median abundance among all proteins in that sample to get the relative protein abundance. Then, the data matrices were automatically scaled as z-score values for subsequent statistical analysis. The pairwise DEPs between TBM, PM, VM, CCM, or BD, and Ctrl were defined by adjusting age and sex as confounding factors by performing a linear modeling limma analysis. Differential expressed proteins (DEPs) were defined as fold change ≥2 or ≤0.5, and along with a Benjamini-Hochberg adjusted p-value <0.05 (Benjamini and Hochberg, 1995). DEPs between TBM and VM were corrected using the Benjamini–Hochberg method. DEPs between TBM and PM, and TBM and CCM, were not corrected. GraphPad Prism (version 9) was used to create scatter plots, box plots, violin plots, and columns for data visualization.

### 2.7 Proposed workflow for biomarker panel selection and evaluation

To generate CSF protein biomarker panels that distinguish TBM patients from other CNS infections (discriminative model, including PM, VM, and CCM) as well as from non-CNS infection patients (diagnostic model), we employed a multilevel screening methodology described in a previous study (Sun et al., 2022) with minor modifications. Firstly, Spearman’s rank correlation was used to assess the intercorrelation among proteins, excluding proteins with a moderately high correlation with more than four or five other proteins (rho ≥ 0.6). Secondly, we utilized proteins with low expression similarity to determine their importance in distinguishing between the two classes in the diagnostic and discriminative models, employing the random Forest R package. To minimize variability, we computed 100 random forests, each consisting of 150,000 trees, to generate averaged mean decrease accuracy values for each protein. The mean decrease accuracy values were averaged across the 100 random forest replicates for each protein. Thirdly, ROC analysis was performed to evaluate the diagnostic performance of the candidate biomarkers. Then, the permutation test was conducted with 1,000 iterations as the performance measure for ROC analysis to select the robust DEPs. Finally, the completeness of each protein in either of the two classification groups was evaluated; only proteins identified in every TBM patient without any imputation and with a completeness of more than 90% completeness in the other groups were retained (**Supplementary Figure S1**).

The biomarker panel evaluation methodology includes internal cross-validation and PCA analysis to evaluate the classification capability of panel proteins. Additionally, *post-hoc* power analyses for Statistical power were calculated for every biomarker in classification models to assess the differentiation capability at the current sample sizes. The receiver operating characteristic (ROC) analysis was performed using the “Biomarker Discovery” module of the MetaboAnalyst 6.0 platform.

### 2.8 Bioinformatic analysis

The Ingenuine Pathway Analysis (IPA) (Krämer et al., 2014) software was used to enrich DEPs to signaling pathways. Log2(FC) of DEPs was used as the observation value for IPA analysis. The p-value of IPA analysis was calculated with the right-tailed Fisher’s exact test and was considered significant if less than 0.05. Protein-protein interaction (PPI) network analysis was conducted utilizing the STRING database (https://string-db.org/) and was visualized through Cytoscape (V.3.8.2) (Franz et al., 2016). The heatmap was rendered using TBtools. Pattern recognition analysis (PCA and OPLS-DA) was administered using SIMCA 14.1 (Umetrics, Sweden) software. Visualization of the hypothetical model was executed utilizing the BioRender platform (https://www.biorender.com).

### 2.9 Cytokine analysis

We classified the 310 cytokines into six types based on the IMMPORT database (Updated: July 2020) (https://www.immport.org/shared/home). The statistical significance of cytokines was determined as they were DEPs in CSF proteomic data. According to a previously published research article (Mo et al., 2024), we matched the association between the 310 cytokines and immune cells. Fifty-three cytokines from our data were involved in the function of multiple immune cells and are highlighted. The shinyCircos (Yu et al., 2018) was used to visualize the proteomic data.

## 3 Results

### 3.1 Study design and clinical characteristics

**Figure 1:**
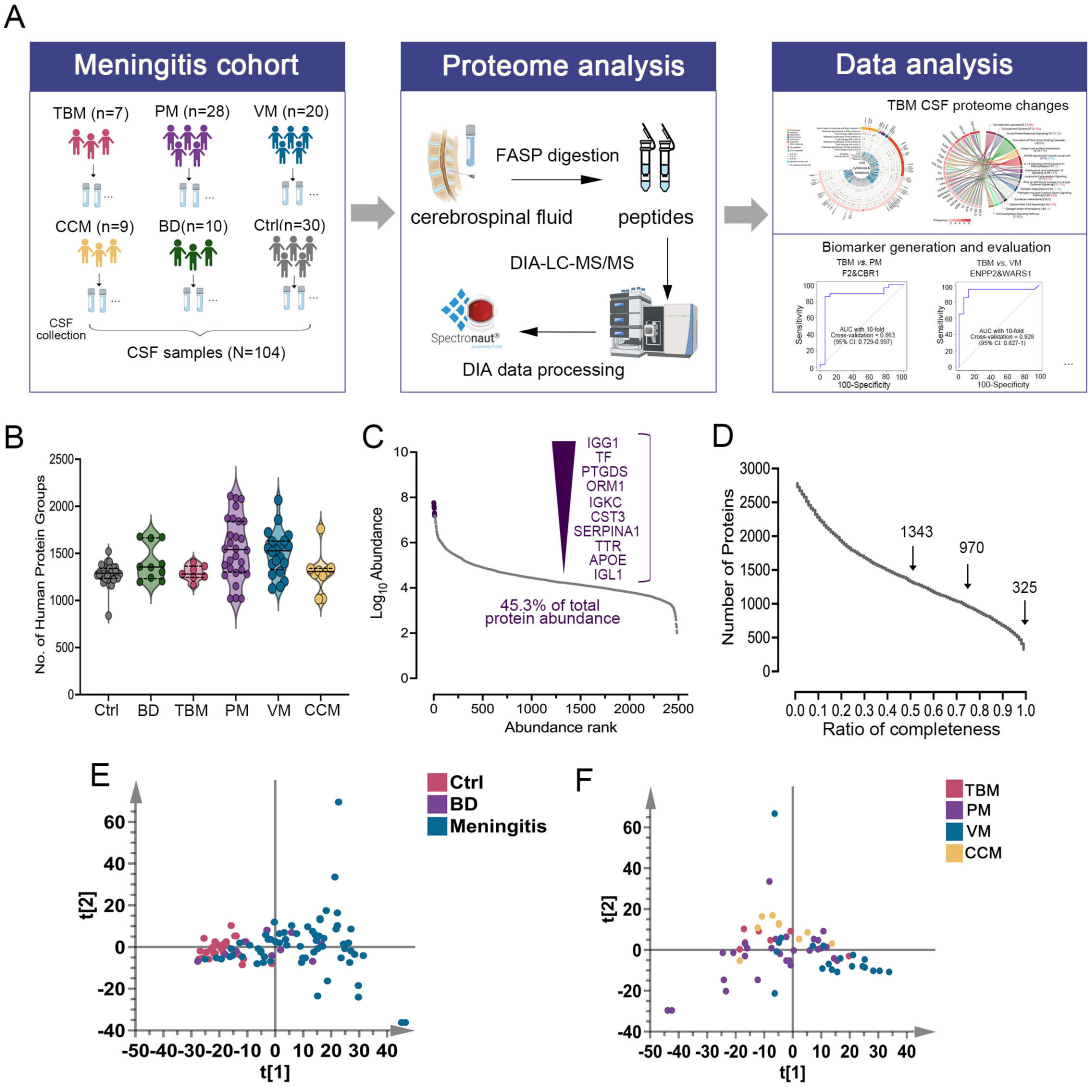
Overview of the CSF proteomics data. **(A)** Overview of the study populations and schematic proteomic workflow. DIA-LC-MS/MS analyzed the CSF of six cohorts. The total number of subjects in each cohort group is depicted. **(B)** Number of proteins identified and quantified with a 1% FDR in six groups. Values are expressed as mean ± standard deviation (SD). **(C)** Proteins in the CSF samples were ranked by median intensity. The top ten abundant proteins are labeled, showing their contribution to the total. **(D)** Data completeness curve. The number of proteins in the dataset (Y-axis) is plotted against the minimum number of samples in which the proteins were quantified (X-axis). Arrows indicate data completeness values of 50%, 75%, and 100%. **(E)** Principal component analysis (PCA) of Ctrl, BD, and Meningitis patients based on their CSF proteome profiles. **(F)** PCA of TBM, PM, VM, and CCM patients based on their CSF proteome profiles.

to systematically identify the characteristic proteomic patterns associated with TBM, we carried out a retrospective DIA-MS-based proteomics workflow (**Figure 1A**). We recruited a total of 104 individuals, who were classified into six groups for DIA-MS quantitative proteomic analysis: TBM (n=7), purulent meningitis (PM, n=28), viral meningitis (VM, n=20), cryptococcus neoformans meningitis (CCM, n=9), non-infectious brain diseases (BD, n=10), and non-CNS infection controls (Ctrl, n=30). The detailed clinical information for these six groups is shown in **Supplementary Table S2**. There were no statistically significant differences in gender (P = 0.44) between these groups. The age distributions were balanced among the TBM, PM, VM, and CM groups, but the Ctrl group samples were younger. The clinical indicators, such as the protein concentration, were higher in TBM than in VM, while the concentrations of glucose and chloride were lower than those in VM, which may reflect the severity differences between TBM and VM (**Table 1**; **Supplementary Figure S2**).

**Table 1:** Baseline demographic and routine laboratory characteristics of CSF among six groups.

Characteristic	Ctrl (n = 30)	BD (n = 10)	TBM (n = 7)	PM (n = 28)	VM (n = 20)	CCM (n = 9)	P1	P2	P3	P4	P5
Gender							0.855	0.1161	0.7976	0.1476	0.7042
Male	16	5	6	16	15	6					
Female	14	5	1	12	5	3					
Age (month)							**0.0008**	**0.0442**	**0.0547**	**0.0003**	**<0.0001**
Median (min-max)	38.24 (13.43-92.10)	96.9 (32.77-163.70)	89.1 (12.37-178.63)	61.35 (12.97-181.63)	89.43 (13.13-161.33)	121.32 (68.80-186.93)					
CSF Parameters											
WBC count (cells/μl)	1.53 (0–8)	58.7 (16-130)	159 (44-340)	676.4 (20-4970)	101.55 (16-342)	200.8 (16-827)	**0.0005**	**<0.0001**	**<0.0001**	**<0.0001**	**<0.0001**
Protein (mg/dL)	203.5 (122-488)	369.7 (194-611)	1269.2 (827-1721)	1125.43 (194-7502)	691.79 (239-4894)	857.8 (310-1511)	**0.0201**	**<0.0001**	**<0.0001**	**<0.0001**	**<0.0001**
Glucose (mg/dL)	3.65 (2.69-5.42)	3.43 (2.76-4.51)	1.9 (1.07-2.83)	2.51 (0.01-7.08)	3.07 (2.42-4.73)	1.7 (0.29-3.77)	0.6077	**<0.0001**	**<0.0001**	**0.0097**	**<0.0001**
Chloride (mg/dL)	124.76 (121.5-135)	124.89 (120.2-127.9)	120.2 (115.5-125.7)	122.46 (111.1-129)	125.23 (110-133.4)	121.4 (116.7-126.7)	0.6045	**0.0135**	0.063	0.3045	**0.0402**
Monocyte count (cells/μl)	0	41.5 (14-72)	133.2 (35-272)	180.11 (12-785)	76.79 (12-325)	78.8 (13-202.5)	**0.0005**	**<0.0001**	**<0.0001**	**<0.0001**	**<0.0001**
polymorphonuclear leukocytes (cells/μl)	0	17.2 (2-104)	25.8 (9-68)	496.57 (2-4771)	14.23 (1-65)	122.1 (3-662)	**0.0002**	**<0.0001**	**<0.0001**	**<0.0001**	**<0.0001**

TBM, Tuberculous meningitis; PM, purulent meningitis; VM, viral meningitis; CCM, *cryptococcus neoformans* meningitis; BD, Brain disease; Ctrl, control group.P1 symbol of BD versus Ctrl, P2 symbol of TBM versus Ctrl, P3 symbol of PM versus Ctrl, P4 symbol of VM versus Ctrl, P5 symbol of CCM versus Ctrl; CSF, Cerebrospinal fluid; WBC, white blood cell. Bold P represent P < 0.05.

Proteins differentially expressed in TBM, PM, VM, and CCM were identified by comparing them to Ctrl and excluding proteins that changed compared to BD. We then used the IPA database for functional annotation to identify key functional terms altered in TBM. Cytokine analysis revealed changes in cytokines and receptors in TBM. Ultimately, we developed diagnostic biomarker panels and evaluated them using receiver operating characteristic (ROC) curves to distinguish TBM from PM, VM, CCM, and Ctrl (**Figure 1A**).

### 3.2 Proteome characterization of CSF samples

We conducted proteome profiling of CSF samples using a high-throughput DIA-MS-based proteomics strategy. By applying this workflow, a total of 2,781 proteins were identified (**Supplementary Table S3**). The average number of protein groups detected for Ctrl, BD, TBM, PM, VM, and CCM groups was 1284, 1405, 1291, 1548, 1503, and 1293, respectively (**Figure 1B**). In all the samples, the quantitative protein intensities across the six groups spanned over six orders of magnitude, and the top ten most abundant proteins accounted for 45.3% of all CSF protein abundance in our datasets (**Figure 1C**). Among the data acquired by DIA, there were 325 proteins with 100% completeness, 970 proteins with 75% completeness, and 1,343 proteins with 50% completeness (**Figure 1D**).

To estimate system stability throughout the entire analysis process, the pooled peptides from all samples were used as a QC to observe the stability of the instrument signal. During the analysis, the QC was analyzed before and after all samples and between every 8–10 samples, resulting in 10 QC samples in our research. To avoid system errors, samples were analyzed in random order, and different groups of samples were interleaved in analysis. The median coefficients of variation (CVs) and correlation coefficients of the QC samples were 0.18 and 0.99 (**Supplementary Figure S3A, B**), demonstrating the consistent stability of the mass spectrometry platform. The PCA analysis of the 10 batches indicated that there was no batch effect in this study (**Supplementary Figure S3C**).

A total of 1429 CSF proteins were retained for further analysis after imputation. Unsupervised principal component analysis (PCA) was conducted among the three groups to visualize the differences in CSF protein profiling among the Ctrl, BD, and meningitis patients. The results indicated a clear separation tendency between the Ctrl and meningitis groups through PCA analysis (**Figure 1E**; **Supplementary Figure S4A**). Despite the unclear separation between the four types of meningitis (**Figure 1F**), the orthogonal partial least squares discriminant analysis (OPLS-DA) mode revealed apparent differences between the four groups (**Supplementary Figure S3C**). One hundred permutation tests confirmed no overfitting of the models (**Supplementary Figures S4B**, **S4D**). Additionally, we assessed the heterogeneity of patients across six groups. The coefficient of variation (CV) values for PM and VM were higher than those of the other groups (**Supplementary Figure S4E**), aligning with the correlation of identified proteins among the six groups and indicating high variability within patients, especially in the PM and VM groups (**Supplementary Figure S4F**). Notably, the TBM and CCM heterogeneity was lower than that of the other two meningitides. The above results demonstrate the potential for discrimination against TBM across different groups.

### 3.3 Cross-comparison of changed CSF proteins between TBM and other CNS infections

**Figure 2:**
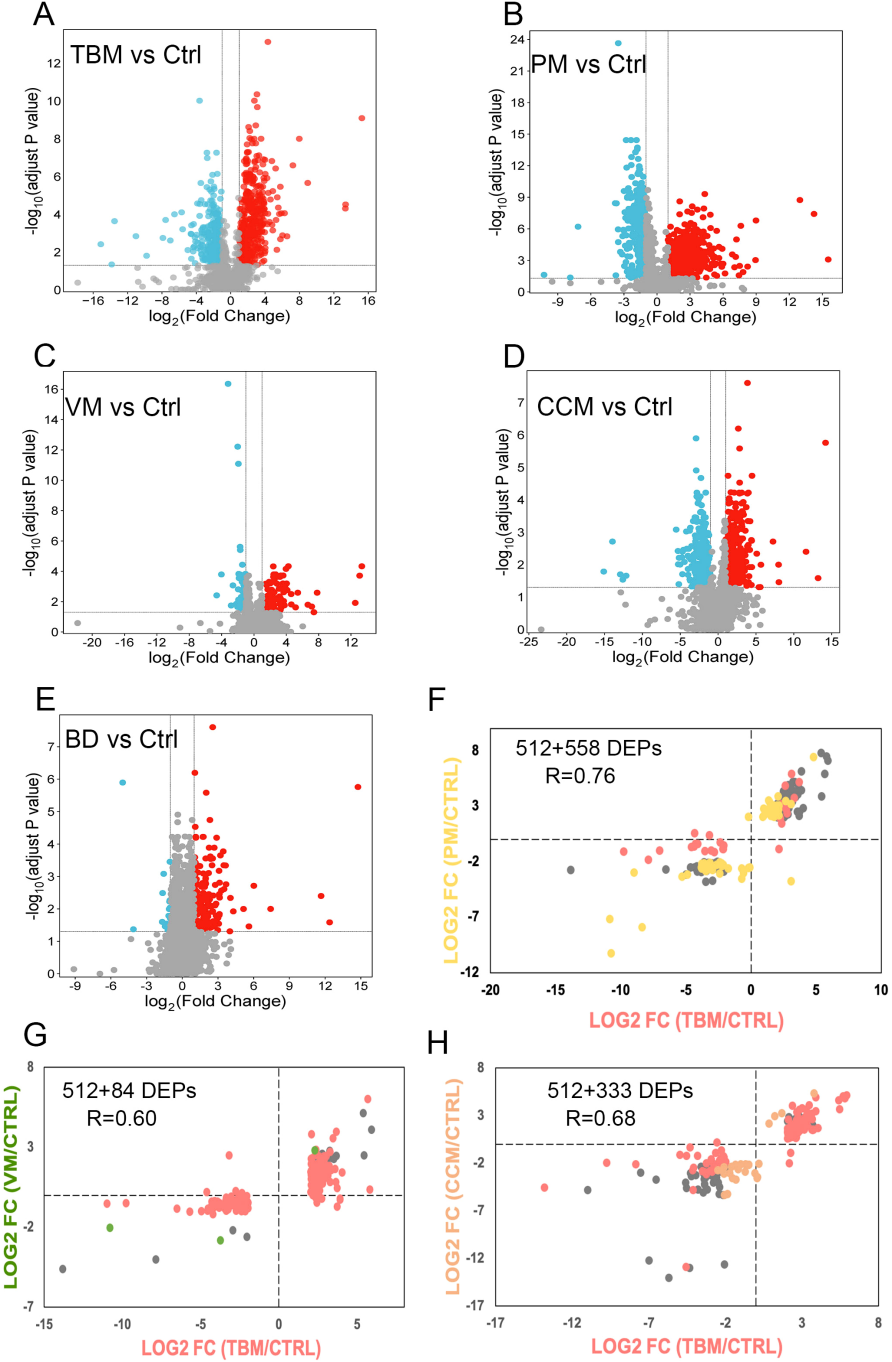
Differential CSF protein selection and comparison. **(A–E)** The volcano plot shows the differentially expressed proteins between TBM **(A)**, PM **(B)**, VM **(C)**, CCM **(D)**, BD **(E)** to Ctrl. The x-axis represents the log2-transformed fold changes of meningitis compared to Ctrl. The y-axis shows the negative log10 of the p-value adjusted by the Benjamini and Hochberg (BH) correction. **(F–H)**. Proteins that were significantly regulated (Benjamini-Hochberg-corrected p-value < 0.05 and log2 fold change of ≧ 2 and ≦ -2) for all three groups were selected. Their fold changes for the two disease groups were plotted against each other: TBM versus PM **(F)**, TBM versus VM **(G)**, and TBM versus CCM **(H)**. The points are colored based on their significance in either one group only (red: TBM; yellow: PM; green: VM; orange: CCM) or in both (grey). Proteins that are absent in one group were plotted at the null line for that group for visualization purposes.

We used linear modeling with limma analysis, adjusting for age and sex as confounding factors, to identify DEPs between TBM vs. Ctrl, PM vs. Ctrl, VM vs. Ctrl, CCM vs. Ctrl, and BD vs. Ctrl. DEPs were defined as fold change ≥2 or ≤0.5, and along with a Benjamini-Hochberg adjusted p-value <0.05. Consequently, a total of 712, 757, 189, 538, and 206 proteins were identified (**Figures 2A–E**), respectively. The changed CSF proteins between BD vs. Ctrl were further excluded to avoid bias. Finally, we selected 512, 558, 84, and 333 DEPs in TBM, PM, VM, and CCM, respectively, for the subsequent cross-comparison analysis (**Supplementary Table S4**).

To evaluate differences in the host response between the sample groups, we plotted the differential proteins in scatter plots, with the corresponding log2 fold change for TBM/CTRL on the x-axis and the log2 fold change for PM/CTRL, VM/CTRL, and CCM/CTRL on the y-axis. The linear regression analysis yielded correlation coefficients of r = 0.76, r = 0.60, and r = 0.68 for the comparisons of TBM and PM, TBM and VM, and TBM and CCM, respectively (**Figures 2F–H**). This cross-comparison reveals that TBM and PM or CCM evoke a more similar response compared to VM.

### 3.4 Distinctive changes of CSF proteome patterns in TBM

We then annotated the canonical pathway of the 512, 558, 84, and 333 proteins in TBM, PM, VM, and CCM patients using the IPA software to compare the altered functional terms in these four CNS infections and identify the characteristic functional terms associated with TBM. As a result, we identified that the complement cascade, coagulation system, complement system, leukocyte extravasation signaling, and acute phase response signaling were prominent due to their strong association and activation levels in TBM patients. In addition, we observed that pathways such as natural killer cell signaling were enriched exclusively in TBM patients (**Supplementary Figure S4A**).

**Figure 3:**
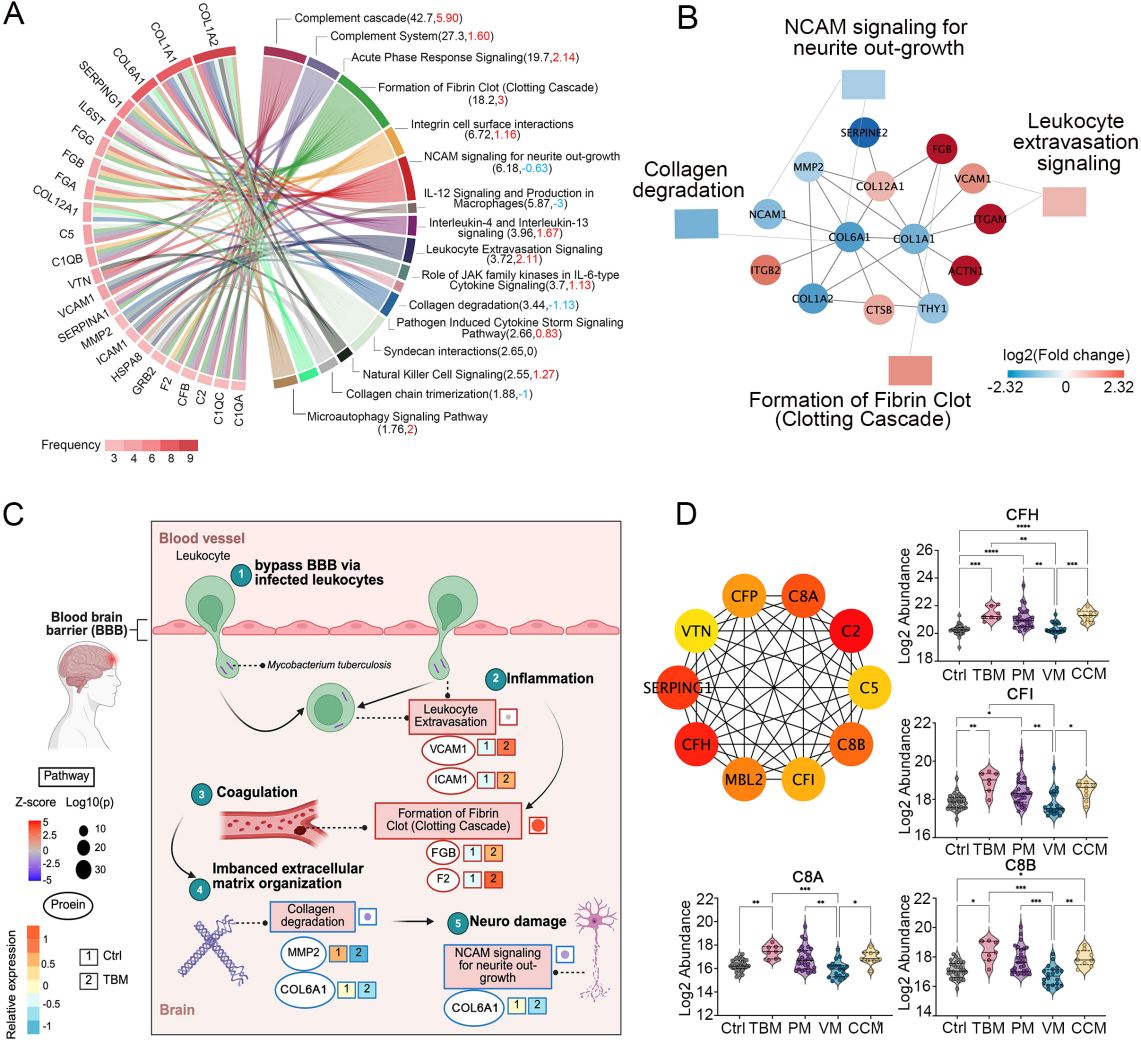
Dysregulated proteins in the CSF of TBM patients. **(A)** The top 23 regulated proteins are ranked by the frequency with which they are found in the 16 representative pathways in TBM, analyzed using Ingenuity Pathway Analysis (IPA). **(B)** The proteins that interacted with COL1A1, COL6A1, COL12A1 and the pathways they participated in. **(C)** Key proteins and pathways characterized in TBM patients in a working model. Proteins involved in leukocyte extravasation, formation of fibrin clot (clotting cascade), collagen degradation, and the NCAM signaling for neurite outgrowth were indicated with their corresponding expression levels in TBM and Ctrl. Created in BioRender. Wei, J. (2025) https://BioRender.com/qc9vxue. **(D)** The top 10 key hub protein interaction networks, along with the relative abundance of regulated proteins in the complement cascade and complement system.

We annotated 16 representative pathways enriched in TBM as they stood out for their association or activation levels, ranking the top 23 regulated proteins by their frequency of enrollment (**Figure 3A**). Notably, collagen proteins COL1A2, COL1A1, and COL6A1 emerged as the most significantly dysregulated proteins. The collagens compose the organized scaffold of the extracellular matrix (ECM) and play crucial roles in the proliferation and differentiation of neuronal progenitors, as well as in dendritic and axonal growth and guidance (Jovanov Milošević et al., 2014; Long and Huttner, 2019). We then conducted a PPI analysis among the regulated proteins involved in the 16 pathways to identify proteins that interact with collagen. We noticed that the COL6A1 interacted with the 72 kDa type IV collagenase (MMP2) and COL1A1. The COL1A1 can interact with vascular cell adhesion protein 1 (VCAM-1) and fibrinogen beta chain (FGB) (**Figure 3B**; **Supplementary Figure S4B**).

The M. tb may bypass barriers via infected macrophages and neutrophils (Davis et al., 2019). Protein VCAM-1 and intercellular adhesion molecule 1 (ICAM-1) are reported to be associated with the inflammation leukocyte extravasation process (Mo et al., 2024), and are upregulated in our proteomic data in TBM patients (**Figure 3C**). In addition, it has been reported that inflammation may initiate coagulation (Esmon, 2005), while in our proteomic data, we identified the upregulated FGB and F2 were enriched in the formation of fibrin clots (clotting cascade). The coagulation factors were reported to interact with the ECM and function as ECM components (Fu et al., 2024). Our proteomic data indicated that the upregulation of coagulation factors, along with the significant downregulation of collagens, may reflect an imbalanced ECM organization. Because the NCAM signaling for neurite outgrowth showed an inhibitory trend (z score = -0.63), and the ECM was reported to play an essential role in neurite outgrowth (Sun et al., 2022), we hypothesized that COL6A1, which is enriched in the NCAM signaling for neurite outgrowth, warrants further functional validation.

We further clustered 22 differential proteins involved in the complement cascade and complement system, as they emerged as the most dominant pathways when compared to PM and CCM (**Supplementary Figure S4C**). The top 10 key hub interactive proteins were analyzed. Notably, CFI, CFH, C8B, and C8A stood out due to their higher fold changes compared to PM and CCM (**Figure 3D**).

Overall, the proteomic data indicate widespread association of the complement cascade, the formation of fibrin clot, the leukocyte extravasation signaling, and the collagen degradation process. The altered proteins in these processes may contribute to the TBM pathogenesis.

### 3.5 Construction of biomarker panels to differentiate TBM from other CNS infections

Based on the selection criteria in the Methods, we conducted a multilevel analysis to define CSF protein signatures to distinguish TBM from PM, VM, CCM, and the non-CNS-infection cohort (Ctrl). First, we selected 15, 17, 11, and 74 DEPs as candidates for feature selection to differentiate TBM vs. PM, TBM vs. VM, TBM vs. CCM, and with the Ctrl group (**Supplementary Figure S1**). To obtain complementary biomarker combinations, we evaluated these proteins using Spearman’s rank correlation. For the differentiation with PM, we excluded 1 protein that had a moderately high correlation with more than four other proteins (rho ≥ 0.6), and the 14 remaining proteins with less interdependency (median correlation coefficient of 0.09) were chosen for subsequent analyses. Similarly, we retained 13 proteins for differentiation with VM, 11 proteins for differentiation with CCM, and 30 proteins for differentiation with Ctrl (**Supplementary Figure S6**).

Next, we evaluated these proteins as input variables and identified the most important features in the discriminative and diagnostic models using the random forest algorithm (**Supplementary Figure S7**). Meanwhile, we measured the classification performance of each protein in the discriminative and diagnostic stratification using ROC analysis (**Supplementary Figure S8A**). The permutation test was conducted with 1,000 iterations as the performance measure for ROC analysis to select the robust DEPs. The ratio that was larger than the original AUC values was calculated as a P-value. Only Proteins with a P-value < 0.05 were retained as candidates for panel construction (**Supplementary Figure S8B**). Furthermore, the completeness of each protein in its corresponding group was also evaluated (**Supplementary Figure S9**). Finally, a CSF protein signature for discrimination with PM consisted of F2 and TRMP; for VM, it included ENPP2 and WARS1; for CCM, it comprised F12, APOM and CD163; and a classifier for TBM diagnosis consisted of HLA-B and MGAT1 was generated, respectively.

**Figure 4:**
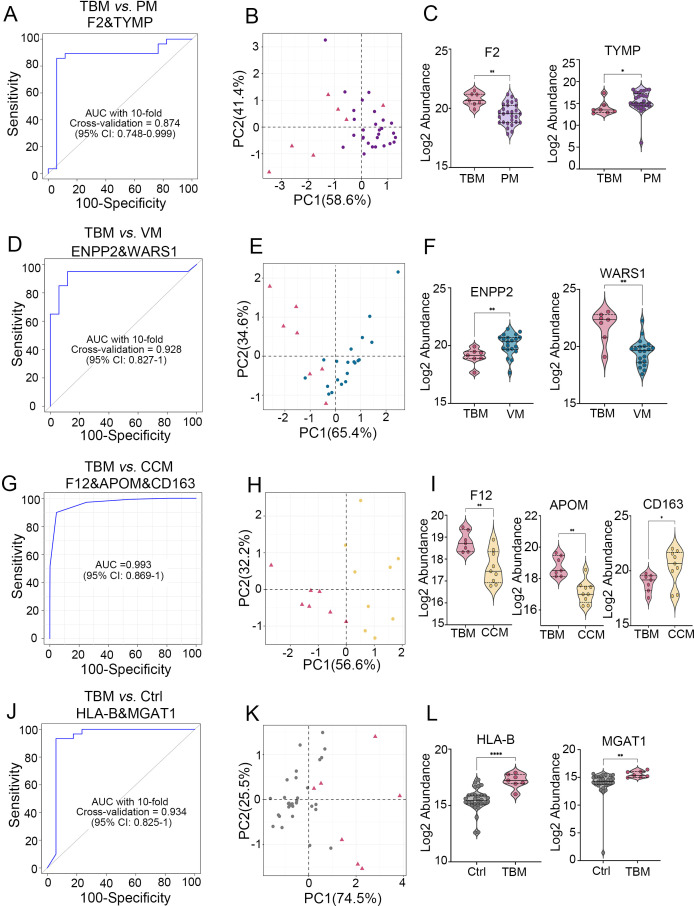
Generation of biomarker combinations to differentiate TBM from other CNS infections and non-CNS infections. **(A)** ROC for the biomarker panel to classify TBM versus PM using 10-fold cross-validation. **(B)** PCA analysis of the two proteins between TBM and PMs. **(C)** Expression pattern of the two proteins between TBM and PM. **(D)** ROC of the biomarker panel for classifying TBM versus VM using 10-fold cross-validation. **(E)** PCA analysis of the two proteins between TBM and VM. **(F)** Expression patterns of two proteins between TBM and VM. **(G)** ROC analysis of the biomarker panel to classify TBM versus CCM using the SVM algorithm. **(H)** PCA analysis of the three proteins between TBM and CCM. **(I)** Protein expression patterns between TBM and CCM. **(J)** ROC for the biomarker panel to classify TBM versus Ctrl using 10-fold cross-validation. **(K)** PCA analysis of the two proteins between TBM and Ctrl. **(L)** Expression patterns of two proteins between TBM and Ctrl.

The panel for classifying TBM and PM achieved an AUC of 0.874 (95% CI, 0.748-0.999) with a specificity of 0.882(95% CI, 0.729-1.0) and a sensitivity of 0.929(95% CI, 0.929-1), as determined by 10-fold cross-validation (**Figure 4A**). Similarly, the panel for classifying TBM and VM achieved an AUC of 0.928 (95% CI, 0.827-1) with a specificity of 0.882(95% CI, 0.729-1.0) and a sensitivity of 0.950(95% CI, 0.950-1), as determined by 10-fold cross-validation (**Figure 4D**). We used the linear SVM algorithm to evaluate the panel classifying TBM and CCM, which achieved an AUC of 0.993 (95% CI, 0.869-1) with a specificity of 1(95% CI, 0.59-1.0) and a sensitivity of 1(95% CI, 0.66-1) (**Figure 4G**). Finally, the TBM diagnostic panel achieved an AUC of 0.934 (95% CI, 0.825-1) with a specificity of 0.941(95% CI, 0.829-1.0) and a sensitivity of 0.933(95% CI, 0.933-1) (**Figure 4J**).

The results of PCA of these biomarker combinations showed clustering of different groups (**Figures 4B, E, H, K**). The normalized expression of each biomarker in different groups is shown in **Figures 4C, F, I, L**. Biomarkers distinguishing TBM from PM showed statistical powers ranging from 0.74 (CBR1) to 0.97 (F2), with values of 0.99 (WARS1) and 0.9 (ENPP2) in differentiating TBM from VM, values of 0.97 (APOM), 0.92 (F12), and 0.76 (CD163) in distinguishing TBM from CCM, and values of 0.99 (HLA-B) and 0.97 (MGAT1) in differentiating TBM from Ctrl. This result indicates that most proteins effectively differentiate patient groups at current sample sizes (**Supplementary Table S5**).

### 3.6 Cytokines and their receptors enrichment analysis in CSF

**Figure 5:**
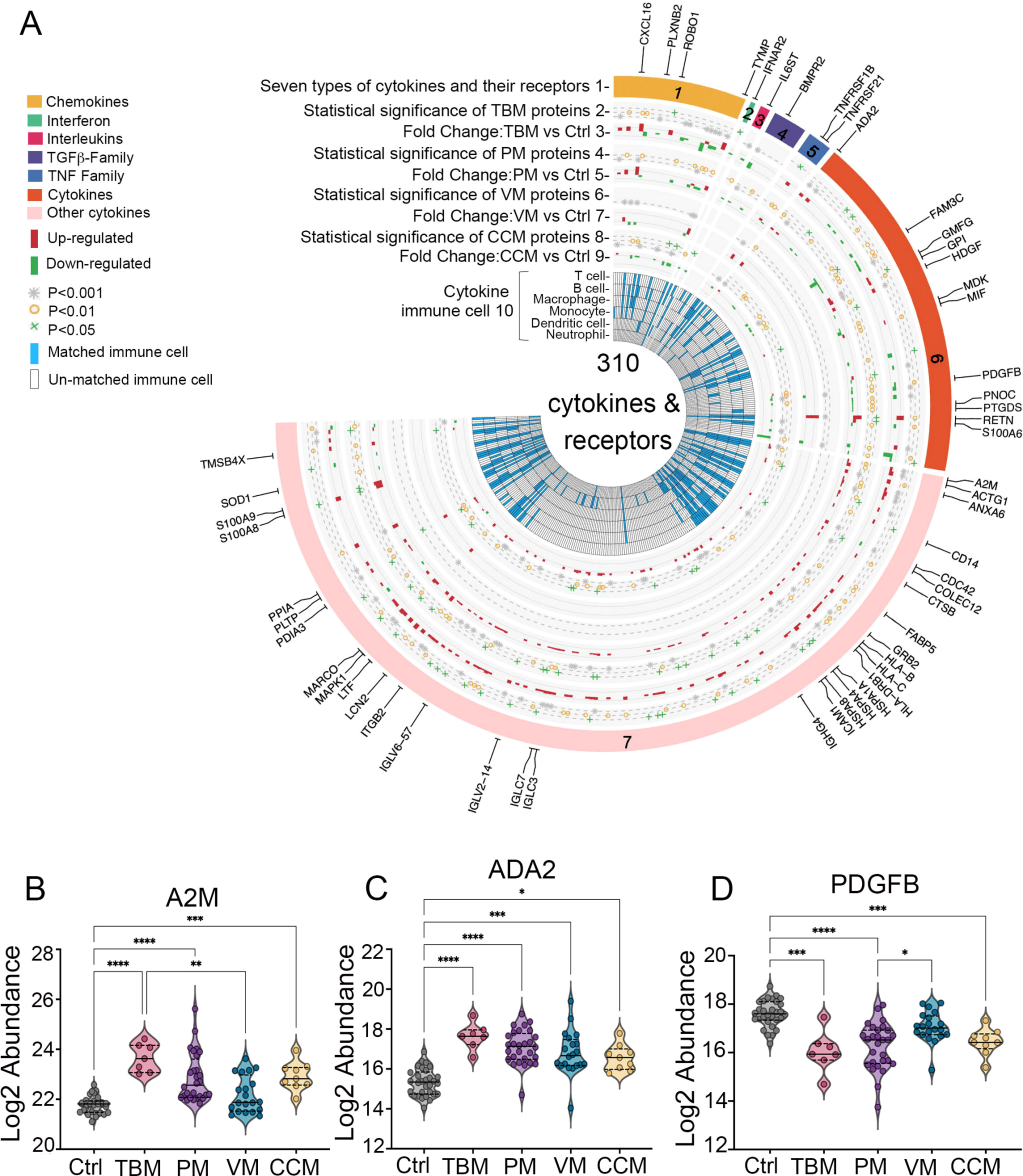
**(A)** Circos plot integrating the relative expression and cytokine-immune cell relationship of 310 cytokines and their receptors. Track 1, the outermost layer, represents 310 cytokines and their receptors, which are organized into six classes. Tracks 2, 4, 6, and 8 identify cytokines from the TBM, PM, VM, and CCM with a cutoff of adjusted p < 0.05 when compared to Ctrl, which were considered statistically significant. Tracks 3, 5, 7, and 9 show the distribution of cytokine abundance in TBM, PM, VM, CCM, and Ctrl. Track 10, the inner circle, illustrates the immune cells associated with each cytokine as reported by previous research. **(B–D)** Expression patterns of A2M, ADA2, and PDGFB in the CSF.

The activated microglia that secrete cytokines may have caused excessive inflammation, contributing to poor outcomes of TBM (Barnacle et al., 2023). In this study, we identified 310 cytokines and their receptors in the CSF. They were categorized into six types: chemokines, interferons, interleukins, transforming growth factor-β (TGF-β) family, tumor necrosis factor (TNF) family, and other cytokines (**Figure 5A**, track 1).

We identified 120, 115, 12, and 68 significantly dysregulated cytokines and receptors from TBM, PM, VM, and CCM, respectively, when compared with the Ctrl group (**Figure 5A**, track 2, 4, 6, 8), totaling 155 significantly dysregulated cytokines and receptors. These modulated cytokines and receptors were enriched for the Complement and coagulation cascades and MAPK signaling pathway (**Supplementary Figure S10**). Most cytokines and receptors in CSF were upregulated (i.e., 91 of 120, 75.8% in TBM; 81 of 115, 70.4% in PM; 7 of 12, 58.3% in VM; 44 of 68, 64.7% in CCM) in meningitis patients compared to Ctrls (**Figure 5A**, track 3, 5, 7, 9).

Cytokines produced by immune cells mediate diverse immune processes. In our data, 53 significantly dysregulated cytokines were involved in the functions of multiple immune cell types (**Figure 1A**, track 10), including 41 in TBM patients, as described in the Materials and Methods. We then focused on the distinct dysregulated cytokines and receptors in TBM. Notably, we identified that A2M and ADA2 exhibited higher levels in TBM than in PM, VM, and CCM when compared to Ctrl, whereas PDGFB displayed the lowest levels in TBM; all were involved in macrophage function (**Figures 5B–D**).

## 4 Discussion

TBM often presents with non-specific symptoms in its early stages and is diagnosed later in the course of the illness when brain damage has already occurred. Early diagnosis and management of TBM are essential, as delays in diagnosis can lead to poor outcomes including death, neurological sequelae, and neurocognitive disorders. In this study, we systematically analyzed the CSF proteome of TBM, PM, VM, and CCM patients, with the aim of identifying distinct changes in the proteome of pediatric TBM. We also included two different control groups: the brain disease control and the non-CNS infected controls, to enhance the accuracy of our results. To date, this is the most comprehensive study to determine the CSF proteome changes and biomarkers in pediatric TBM.

We observed that various distinctive pathways were enriched in pediatric TBM. For instance, the activation of pathways such as the complement cascade (Z score = 5.90) and the formation of fibrin clot (Z score = 3) were most significant compared to PM, VM, and CCM. The complement system is a tightly regulated innate immune mechanism that plays a crucial role in normal central nervous system (CNS) development and function (Fatoba et al., 2022). Previous research indicated that complement-activated microglial Macro_C01 cells are linked to a neuroinflammatory response that results in persistent pediatric TBM meningitis (Mo et al., 2024), highlighting the important roles of complement activation in TBM pathogenesis. Notably, we found that CFI, CFH, C8B, and C8A not only acted as key interacting proteins in the complement system but also exhibited higher levels than PM and CCM, indicating their significant contribution to TBM. CFB participates in the complement alternative pathway, while CFI helps to sequentially cleave C3b into inactivated C3b (iC3b) and C3d, leading to the complete inactivation of C3 and C5 convertases, evading homologous attacks via the alternative pathway (Dalakas et al., 2020). C8A and C8B are components of the membrane attack complex (MAC). These results suggest that anti-complement therapeutic strategies targeting molecules in complement activation may serve as a potential approach for future pediatric TBM treatment. Fibrin clot formation represents the final step in the coagulation pathway (Gerstman et al., 2023), and the hypercoagulable state observed in childhood TBM is comparable to that described in adults with pulmonary tuberculosis, possibly increasing the risk of infarction (Schoeman et al., 2007). Therefore, therapeutic measures that reduce the risk of thrombosis could be potentially beneficial in childhood TBM.

Notably, we observed that collagen proteins, including COL1A2, COL1A1 and COL6A1 were downregulated and emerged as key participants in the most distinctive pathways in TBM. Ablation of COL6A1 has been reported as a direct link with defective dopaminergic activity, through a mechanism involving the inability of meningeal cells to sustain dopaminergic differentiation, indicating its related neurobehavioral features in both mice and humans (Gregorio et al., 2022). Since the COL6A1 also enriched in the NCAM signaling for neurite outgrowth pathway, its biological mechanism in TBM pathogenesis worth future casual validation.

A total of 9 CSF proteins in TBM were selected to differentiate between PM, VM, CCM, and Ctrl, respectively. Some of these proteins have been reported to be associated with the pathogenesis of brain diseases. For example, F2 and TYMP were generated to distinguish among PM. F2 is involved in fibrin clot formation, which may increase the risk of infarction, as we discussed previously. ENPP2 was selected to differentiate from VM and is reported to be strongly up-regulated in reactive astrocytes adjacent to the lesion following neurotrauma (Savaskan et al., 2007). APOM was in the TBM and CCM classification panel and was reported as potential diagnostic biomarkers of pediatric bacterial meningitis (Luo et al., 2022). MGAT1 has been reported to be highly expressed in glioblastoma and promotes glioma cells partly through the upregulation of Glut1 protein (Li et al., 2020). These results demonstrate that the biomarker panels selected in our research are associated with the pathogenesis of TBM to some degree.

The host inflammatory response significantly influences TBM pathology (Rohlwink et al., 2019), with many sequelae linked to a dysregulated immune response. Thus, effective host-directed therapies (HDT) are critical for improving TBM survival and outcomes. From the IMMPORT database, we identified 120 dysregulated cytokines and receptors in TBM, with 41 affecting multiple immune cell types. Notably, 35 were upregulated compared to controls, particularly A2M and ADA2, which were more upregulated than in other meningitis types. A2M, a key glycoprotein, regulates proteolysis and supports cell migration while binding cytokines and damaged proteins (Vandooren and Itoh, 2021). Plasma levels of A2M-containing microparticles are higher in sepsis survivors than nonsurvivors or healthy volunteers (Dalli et al., 2014). A2M abundance also correlates with inflammation in rheumatic diseases (Flory and Vischer, 1981; Ekerot and Ohlsson, 1982) and may serve as a diagnostic biomarker for pediatric BM (Luo et al., 2022). Although A2M is associated with sepsis outcomes and has diagnostic potential in pediatric BM, we found that A2M levels in TBM were higher than in PM, VM, and CCM. A2M binds several cytokines, including PDGF (Rehman et al., 2013), aligning with the downregulation of PDGFB in our findings. CSF ADA levels help differentiate TBM from other meningitis types in adults (Cho et al., 2013; Song et al., 2022; Chaurasia et al., 2023; Pannu et al., 2023). Our data show ADA2 levels are elevated in pediatric TBM compared to PM, VM, and CCM. We propose that targeting A2M and ADA2 may be a viable HDT adjunct therapy for pediatric TBM, pending validation in animal models or clinical studies.

This study used an unbiased DIA-MS-based proteomics workflow to identify biomarker panels and validate them through internal cross-validation; further external or temporally separated validation is necessary to assess its generalizability. The targeted parallel reaction monitoring (PRM) assays provide precise quantitative analysis of specific proteins by selectively monitoring target peptide ions during MS analysis. PRM is antibody-free, multiplexable, highly sensitive, and reproducible (Kulyyassov et al., 2021), which assists in narrowing down candidate DEPs for biomarker development and identifying the most suitable proteins for further immunoassay validation in a larger cohort. Although instrumentation costs are higher, per-sample expenses for multi-target analysis are comparable. Turnaround time for the PRM analysis is about one week (including method optimization), with further improvements expected through next-generation mass spectrometers and automated sample preparation (Barkovits et al., 2021). After passing the PRM validation, the turnaround time for immunoassays on targeted panels is about 1–3 days. Clinical applications demonstrate the strong concordance with results between PRM and ELISA (Martinez-Garcia et al., 2017). The PRM-ELISA-based validation strategy would be appropriate in future analysis.

Previous research used immunoassays to validate the accuracy of recently identified host 3-biomarkers (VEGF-A/IFN-γ/MPO) and 4-biomarkers (MPO/IFN-γ/ICAM1/IL-8), and constructed a new combination panel (CC4b/CC4/CCL1/procalcitonin) with an AUC of 0.98 (95% CI, 0.94–1.00) as the diagnostic candidates for childhood TBM (Manyelo et al., 2022). Notably, protein ICAM1 was also identified as a DEP in our proteomic data from TBM patients, indicating the potential for validating our panels using immunoassays in future analyses.

The *post-hoc* power analysis was also performed to evaluate the fold change of the top ten up-regulated DEPs across the four comparisons using age- and sex-adjusted DEPs (TBM vs Ctrl, PM vs Ctrl, VM vs Ctrl, and CCM vs Ctrl). The statistical powers ranged from 0.48 to 0.99 (TBM vs Ctrl), 0.51 to 0.98 (PM vs Ctrl), 0.23 to 0.99 (VM vs Ctrl), and 0.68 to 0.99 (CCM vs Ctrl) (**Supplementary Table S5**). Despite some limited low discriminatory powers (MAP2, power = 0.23; ROBO1, power = 0.48), the average discriminatory power was high, reaching 0.84, 0.82, 0.875, and 0.91 in distinguishing TBM, PM, VM, and CCM from Ctrl.

Key limitations include the small cohort size and single-center retrospective design. Though we performed a strict biomarker selection procedure and validated it using internal cross-validation, external or temporally separated validation is necessary to assess its generalizability. Additionally, the hypotheses proposed in this research were solely based on proteomic associations, which need further causal validation.

## 5 Conclusion

In summary, we utilized an unbiased proteomic strategy to reveal distinctive proteome changes in pediatric TBM patients. The TBM proteome exhibited greater similarity to that of PM patients. A2M, ADA2, and PDGFB, as cytokine receptors, displayed higher or lower levels compared to the other three meningitis types. The altered CSF proteins were more associated with the complement cascade and fibrin clot formation. A series of combination protein panels was selected using stringent criteria to differentiate TBM from PM, VM, CCM, and non-CNS-infected patients. Ultimately, this research enhances our biological understanding of TBM, providing insights into valuable differential diagnostic biomarkers and potential therapeutic targets for pediatric TBM in the future. Future research should focus on validation through larger, multi-institutional studies to ensure robustness and generalizability across various clinical settings.

## Data Availability

The datasets presented in this study can be found in online repositories. The names of the repository/repositories and accession number(s) can be found in the article/**Supplementary Material**.
