# Supplementary material for: Proteomic profiling of cerebrospinal fluid uncovers distinctive pathophysiological changes and potential biomarkers in pediatric tubercular meningitis
Source: Front Cell Infect Microbiol. 2025 Oct 16;15:1662783. doi: 10.3389/fcimb.2025.1662783 (PMC12571815; doi:10.3389/fcimb.2025.1662783)
Supplement: Supplementary file 1 [file DataSheet1.pdf]

Proteomic profiling of cerebrospinal fluid uncovers distinctive pathophysiological changes and potential biomarkers in pediatric tubercular meningitis

Jing Wei[1†], Liang Zhu[2,3†], Binglin Jian[2,3], Yuncui Yu[1], Bing Hu[2,3], Lingyun Guo[2,3], Huili Hu [2,3], Zhenzhen Dou [2,3], Linlin Liu [2,3], Gang Liu[2,3*], Peng Guo[1,4*]

[1]Clinical Research Center, Beijing Children's Hospital, Capital Medical University, National Center for Children's Health, Beijing, China.

[2]Department of Infectious Diseases, Key Laboratory of Major Diseases in Children, Ministry of Education, Beijing Children's Hospital, Capital Medical University, National Center for Children's Health, Beijing, China.

[3]Research Unit of Critical Infection in Children, Chinese Academy of Medical Sciences, Beijing 2019RU016, Beijing, China.

[4]Key Laboratory of Major Diseases in Children, Ministry of Education, Beijing Children's Hospital, Capital Medical University, National Center for Children's Health, Beijing, China.

*** Correspondence:**
Gang Liu, Peng Guo
liugangbch@sina.com; guopeng@bch.com.cn
†These authors contributed equally to this work.

Supplementary Information

Supporting Information

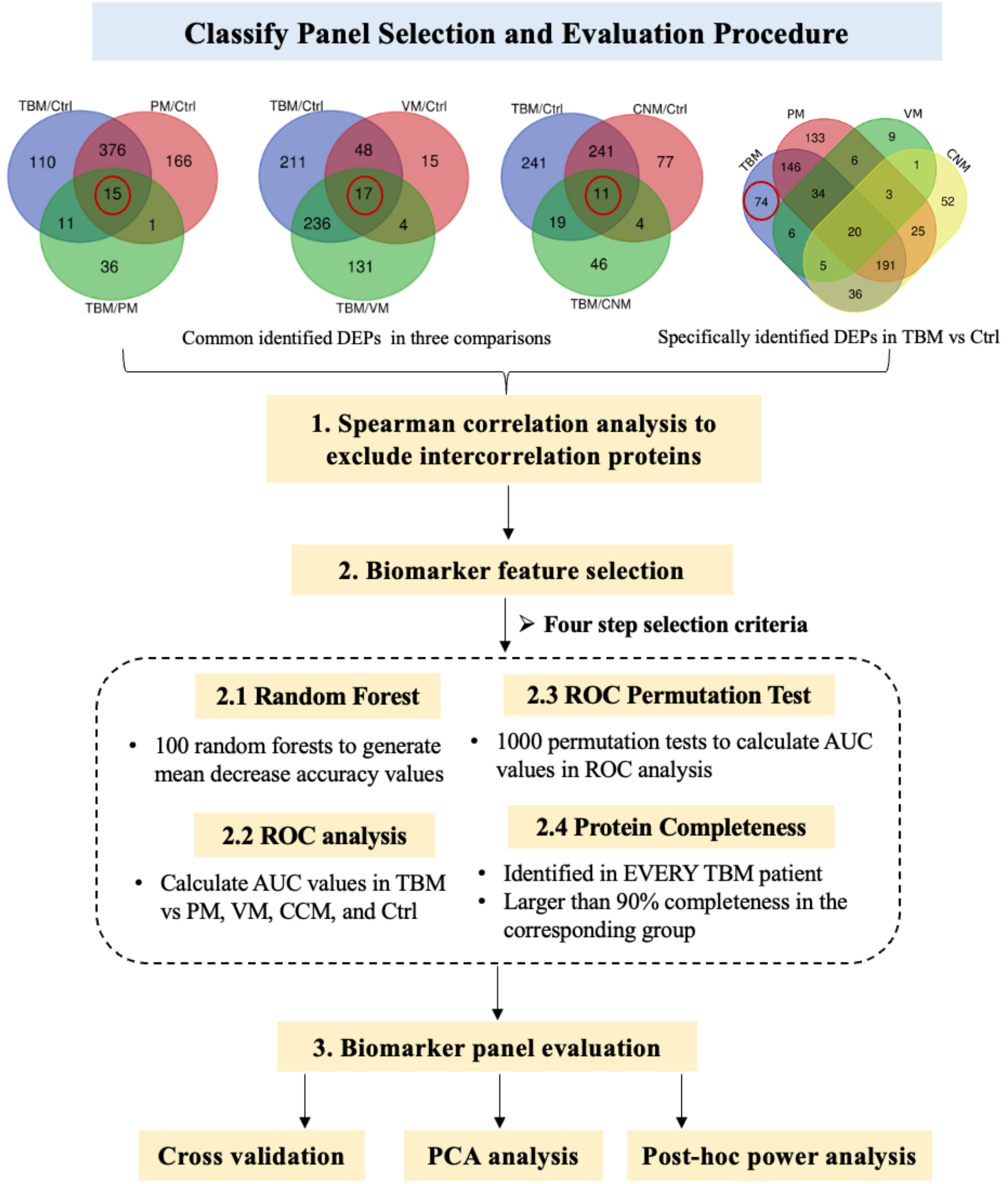

Figure S1. The schematic diagram depicts the suggested workflow for choosing and assessing the biomarker panel.

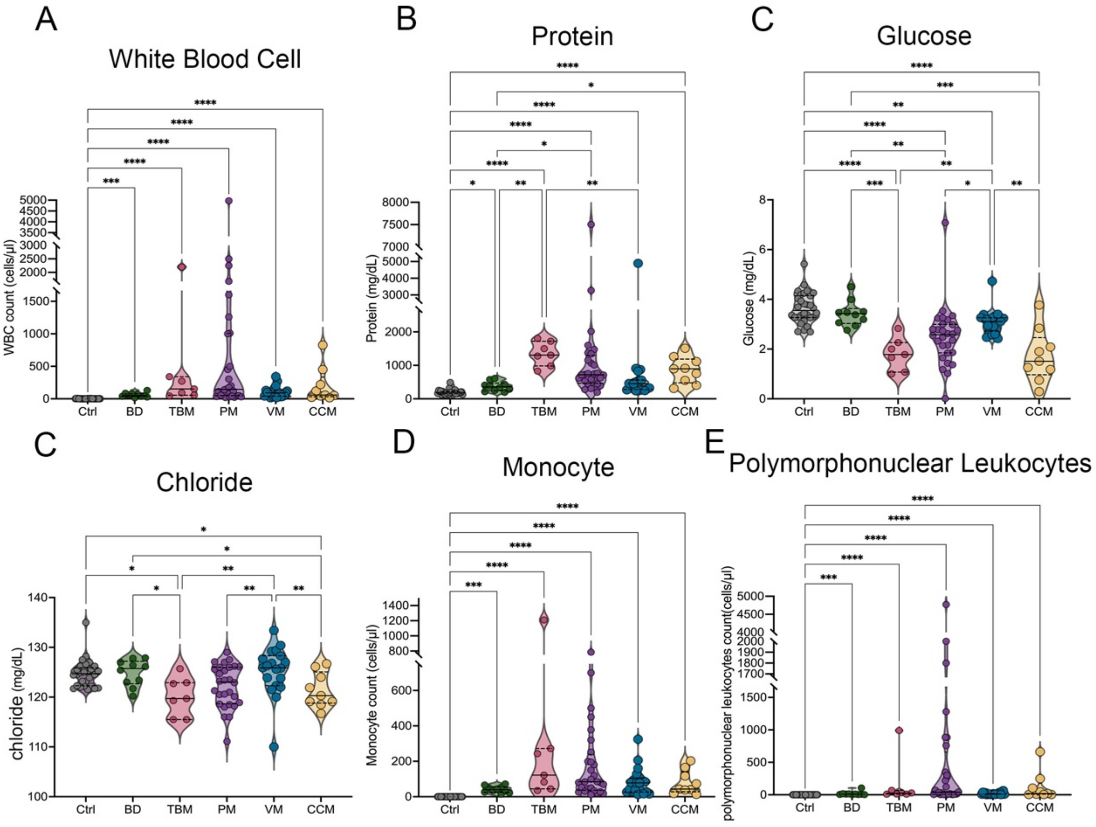

Figure S2. Clinical indicators comparison in the six groups.

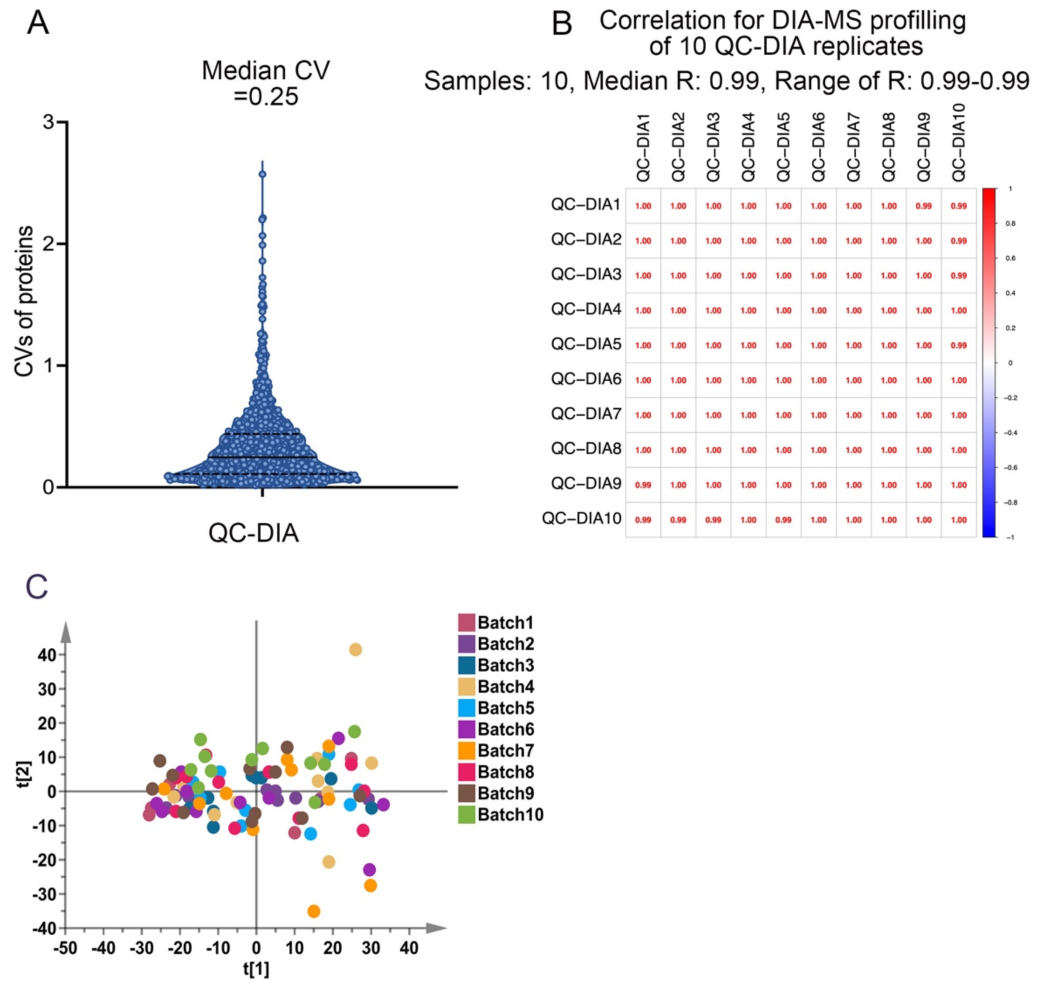

Figure S3. (A) Distribution of CVs of standards in QC-DIA (n = 10). Data represent median, 25% quartile and 75% quartile; (B) The Pearson's correlation coefficients of QC-DIA samples (n = 10 independent experiments)**.** (C) Score plot of unsupervised principal component analysis (PCA) overview of CSF proteomics among the ten batches for LC-MS/MS analysis.

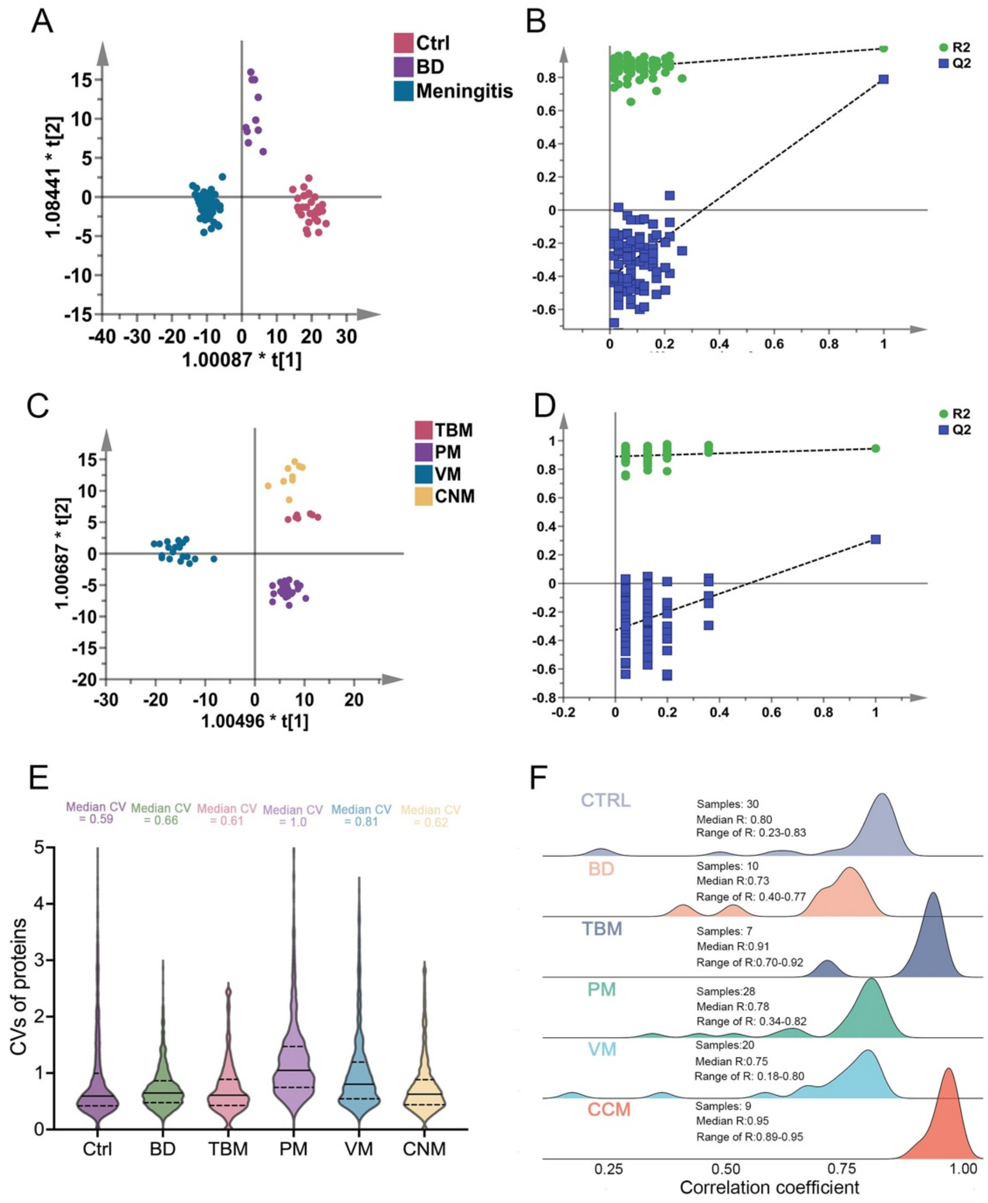

Figure S4. CSF proteome patterns in meningitis and control group. (A) Score plot of orthogonal partial least squares discriminant analysis (OPLS-DA) model among the three groups. (B) One hundred permutation validations of the OPLS-DA model based on the proteome of the three groups. (C) Score plot of orthogonal partial least squares discriminant analysis (OPLS-DA) model among the four groups. (D) One hundred permutation validations of the OPLS-DA model based on the proteome of the four groups. (E) Distribution of CVs of CSF samples in six groups (Ctrl, n = 30 biologically independent samples; BD, n = 10 biologically independent samples; TBM, n =7 biologically independent samples; PM, n = 28 biologically independent samples; VM, n = 20 biologically independent samples; CCM, n = 9 biologically independent samples). Data represent median, 25%, and 75% quartile. (F) The Pearson's correlation coefficient distribution of identified proteins in six groups (Ctrl, n = 30 biologically independent samples; BD, n = 10 biologically independent samples; TBM, n =7

biologically independent samples; PM, n = 28 biologically independent samples; VM, n = 20 biologically independent samples; CCM, n = 9 biologically independent samples).

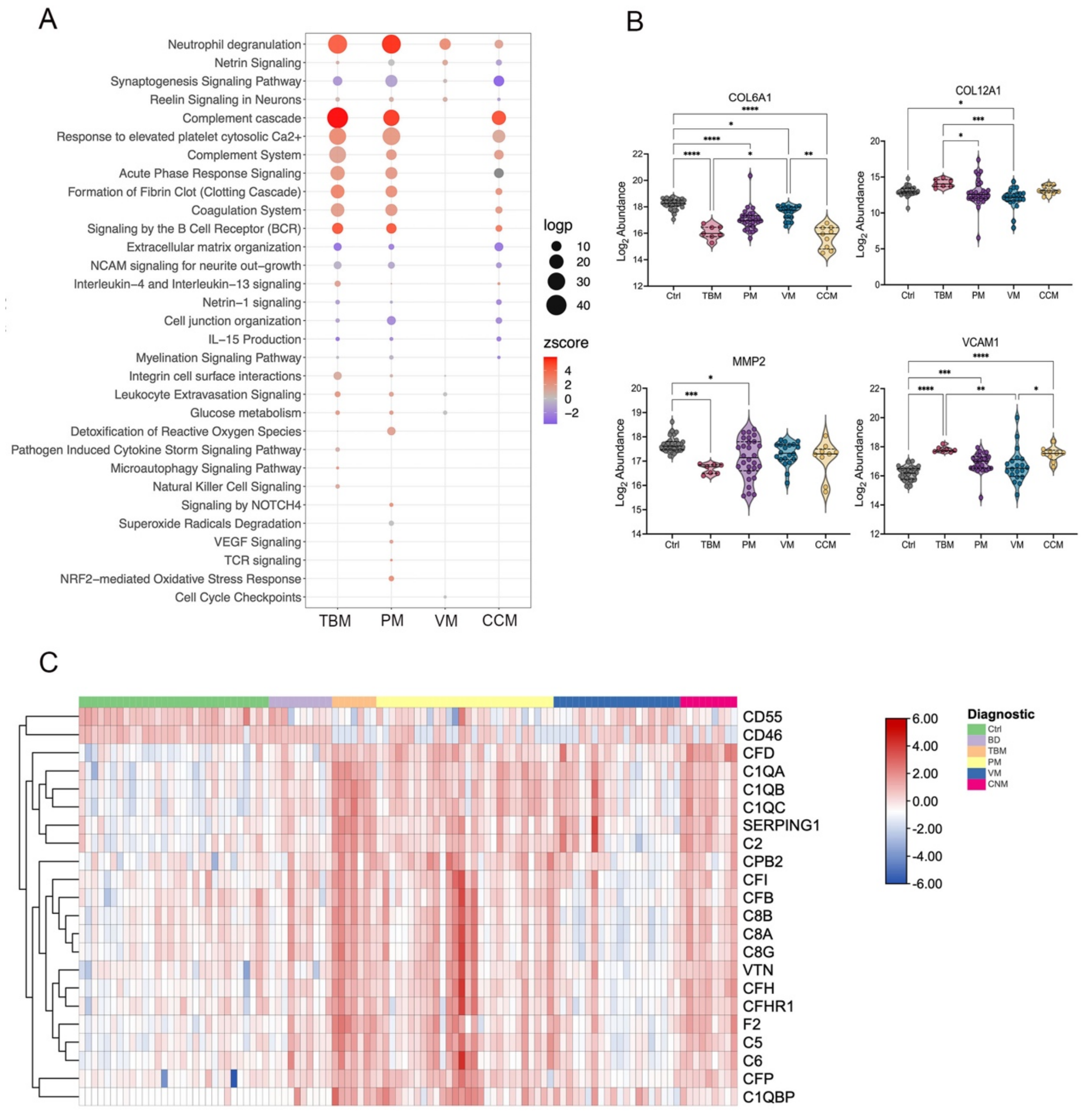

Figure S5. Functional analysis of differential proteins in the TBM, PM, VM and CCM. (A) Representative pathways of differential proteins in the TBM, PM, VM and CNM. The z-score algorithm was used to predict the activation state (either activated or inhibited) of the pathways. If the z-score ≤ − 2, the pathway is predicted to be significantly inhibited. (B) Expression patterns of COL6A1, COL12A1, MMP2, and VCAM1 in the CSF. (C) Unsupervised clustering analysis of 22 deregulated proteins involved in the complement cascade and the complement system.

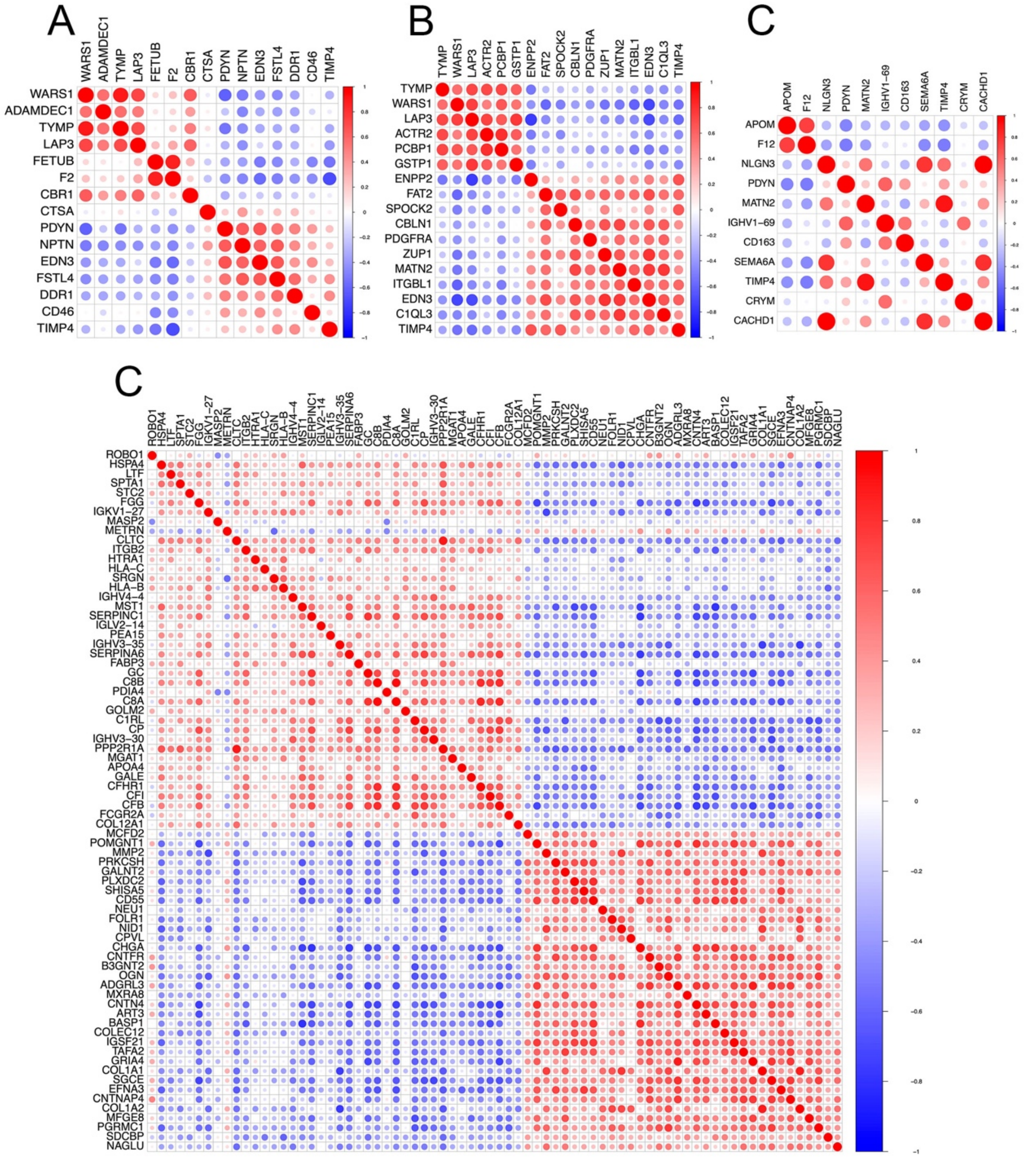

Figure S6. The correlation analysis of the CSF protein biomarker panel signature. (A) The correlation matrix plot of 15 dysregulated proteins to discriminate TBM vs PM. (B) The correlation matrix plot of 17 dysregulated proteins to discriminate TBM vs VM. (C) The correlation matrix plot of 11 dysregulated proteins to discriminate TBM vs CNM. (D) The correlation matrix plot of 74 dysregulated proteins to discriminate TBM vs Ctrl.

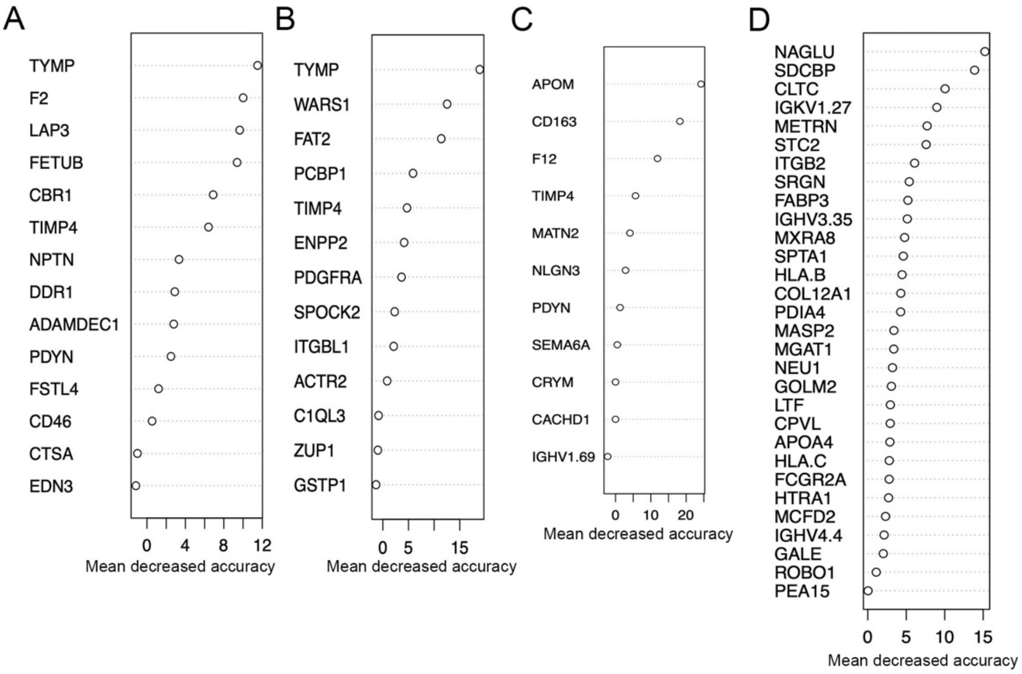

Figure S7. Variable importance plots produced by the random forest algorithm are measured as each variable's mean decrease in accuracy. (A) The important predictors to discriminate TBM vs PM. (B) The important predictors to discriminate TBM vs VM. (C) The important predictors to discriminate TBM vs CCM. (D) The important predictors to discriminate TBM vs Ctrl. The most important predictors have the highest mean decrease accuracy values.

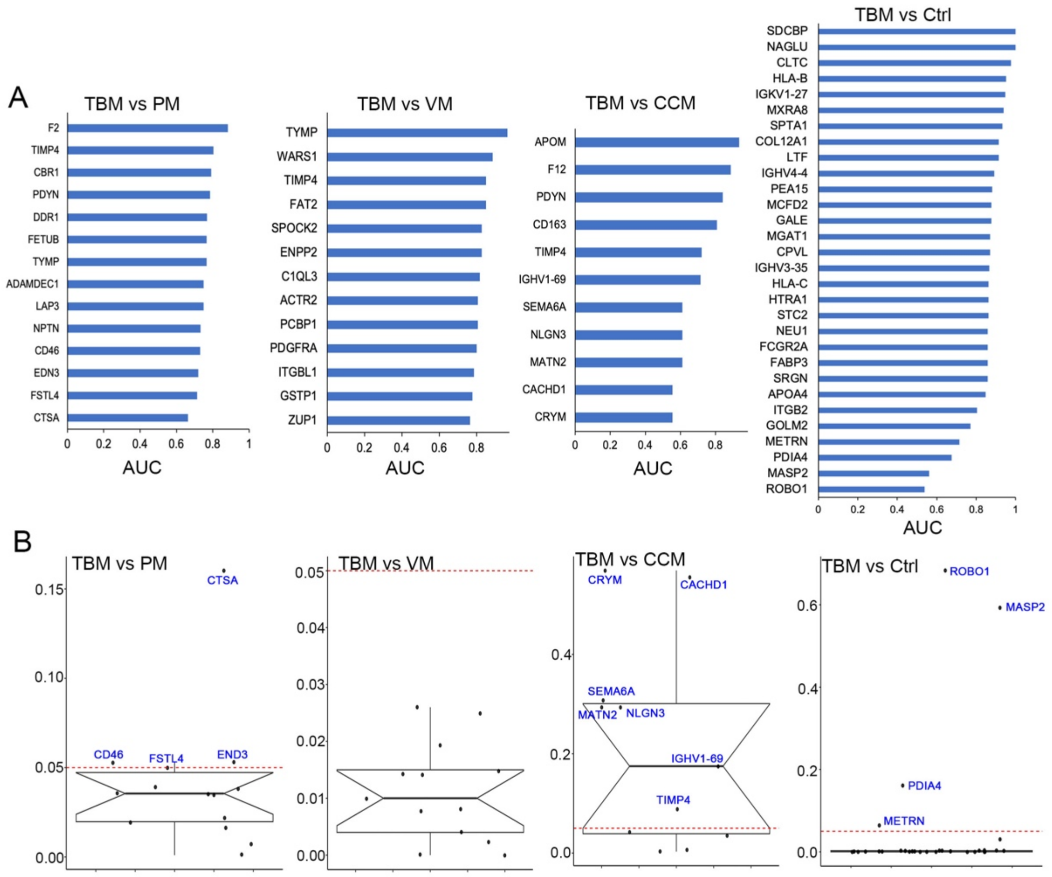

Figure S8. The ROC and permutation analysis. (A) The AUC was used to evaluate the ability of individual proteins to distinguish TBM between PM, VM, CCM and Ctrl. (B) P values of 1000 permutation test to evaluate ROC performance.

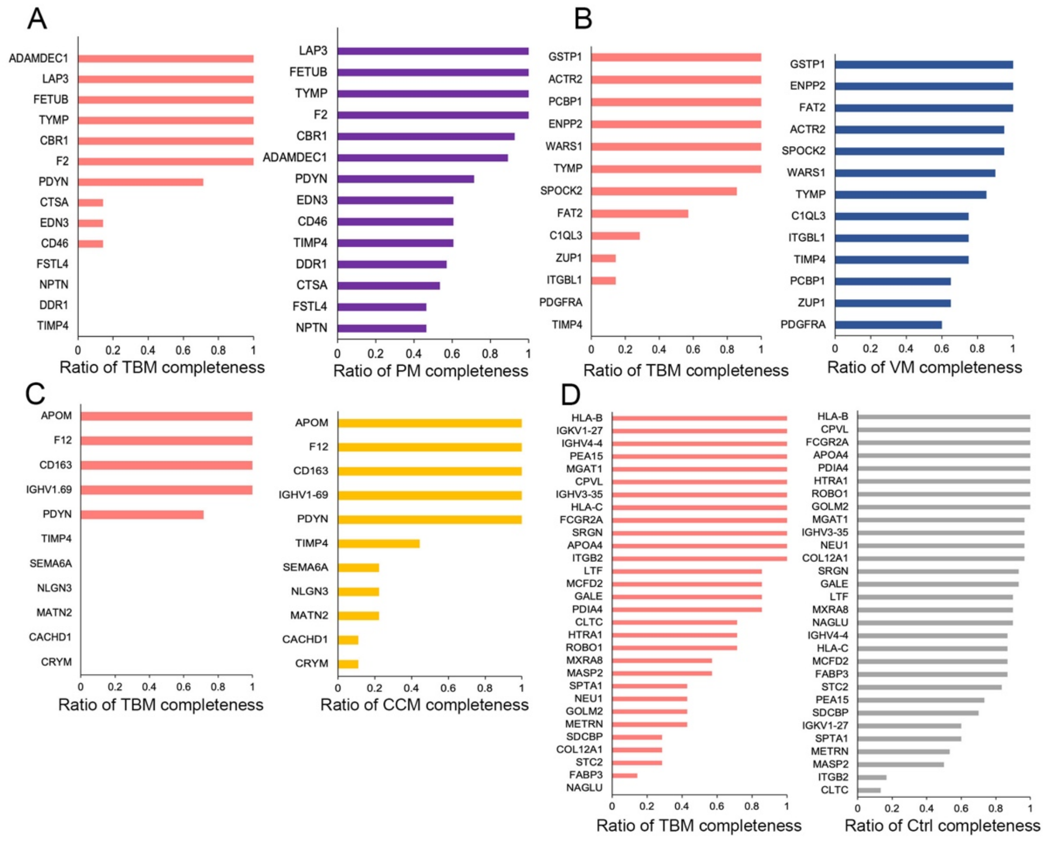

Figure S9. The completeness analysis of the CSF protein biomarker panel signature. (A) The completeness of each protein for discriminating between TBM and PM in their corresponding group. (B) The completeness of each protein for discriminating between TBM and VM in their corresponding group. (C) The completeness of each protein for discriminating between TBM and CCMin their corresponding group. (D) The completeness of each protein for discriminating between TBM and Ctrl in their corresponding group.

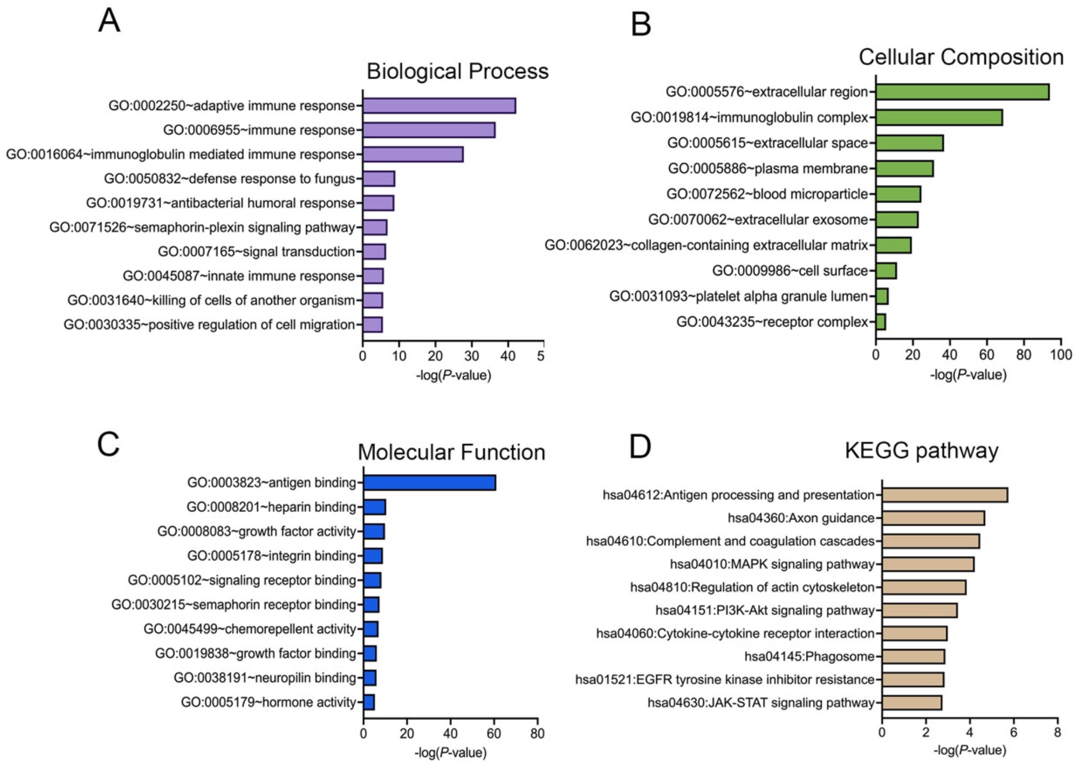

Figure S10. GO analysis of these 155 significantly dysregulated cytokines and receptors. (A) Biological process; (B) Cellular composition; (C) Molecular Function; (D) KEGG pathway.

Table S1. The variable isolation windows for DIA acquisition.
Table S2. The detailed clinical information of enrolled patients.
Table S3. The identified protein groups in this research.
Table S4. Differential proteins in TBM, PM, VM and CCM compared with Ctrl after age and sex adjustment.
Table S5. The power analysis results of the top10 upregulated DEPs and proteins in biomarker panels.
